# Enoyl coenzyme a hydratase 1 attenuates aortic valve calcification by suppressing Runx2 via Wnt5a/Ca^2+^ pathway

**DOI:** 10.1002/ccs3.12038

**Published:** 2024-05-31

**Authors:** Caijun Rao, Baoqing Liu, Haojie Qin, Zhipeng Du

**Affiliations:** ^1^ Department of Geriatrics Tongji Hospital Tongji Medical College Huazhong University of Science and Technology Wuhan China; ^2^ Department of Cardiovascular Surgery Union Hospital Tongji Medical College Huazhong University of Science and Technology Wuhan China; ^3^ Clinic Center of Human Gene Research Union Hospital Tongji Medical College Huazhong University of Science and Technology Wuhan China; ^4^ Department of Cardiology Union Hospital Tongji Medical College Huazhong University of Science and Technology Wuhan Hubei China; ^5^ Department of Gastroenterology Institute of Liver and Gastrointestinal Diseases Tongji Hospital Tongji Medical College Huazhong University of Science and Technology Wuhan China

**Keywords:** aortic valve calcification, ECH1, Runx2, Wnt5a/Ca^2+^pathway

## Abstract

The morbidity and death rates of calcified aortic valves|calcific aortic valve (CAV) disease (CAVD) remain high for its limited therapeutic choices. Here, we investigated the function, therapeutic potential, and putative mechanisms of Enoyl coenzyme A hydratase 1 (ECH1) in CAVD by various in vitro and in vivo experiments. Single‐cell sequencing revealed that ECH1 was predominantly expressed in valve interstitial cells and was significantly reduced in CAVs. Overexpression of ECH1 reduced aortic valve calcification in ApoE^−/−^ mice treated with high cholesterol diet, while ECH1 silencing had the reverse effect. We also identified Wnt5a, a noncanonical Wnt ligand, was also altered when ECH1 expression was modulated. Mechanistically, we found that ECH1 exerted anti‐calcific actions through suppressing Wnt signaling, since CHIR99021, a Wnt agonist, may significantly lessen the protective impact of ECH1 overexpression on the development of valve calcification. ChIP and luciferase assays all showed that ECH1 overexpression prevented Runx2 binding to its downstream gene promoters (osteopontin and osteocalcin), while CHIR99021 neutralized this protective effect. Collectively, our findings reveal a previously unrecognized mechanism of ECH1‐Wnt5a/Ca^2+^ regulation in CAVD, implying that targeting ECH1 may be a potential therapeutic strategy to prevent CAVD development.

## Graphical abstract

ECH1 attenuated aortic valve calcification by suppressing Runx2 via Wnt5a/Ca^2+^ pathway. Overexpression of ECH1 reduced aortic valve calcification, while CHIR‐99021, a Wnt agonist, may significantly lessen the protective impact of ECH1 overexpression on the development of valve calcification.

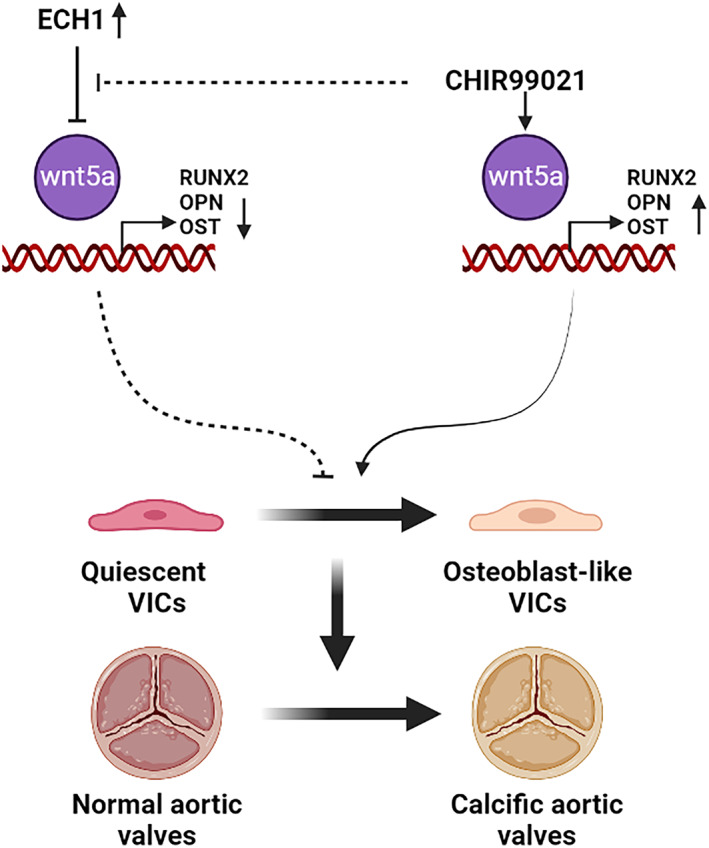

## 1 INTRODUCTION

Calcified aortic valves|calcific aortic valve (CAV) disease (CAVD) is the most common valvular heart disease worldwide, with substantial morbidity and mortality, and it is progressively affecting the elderly.^1^ There is currently no pharmaceutical therapy for CAVD. Although surgical and transcatheter aortic valve replacement remain the most efficient therapeutic options, they frequently result in complications and poor long‐term prognosis. As a result, research into alternative therapy choices is continuing. The essential feature of valvular calcification is the phenotypic transformation of human aortic valve interstitial cells (hVICs) to an osteoblast‐like phenotype.^2^, ^3^ Therefore, efficient approaches to inhibit hVICs osteogenic development may offer novel therapeutic options for CAVD.

Aortic valve calcification is a dynamic process involving mechanical and metabolic variables that results in valve degeneration and stenosis.^4^, ^5^ The key aspects of aortic valve calcification that require VICs activation to osteoblast and bone formation have sparked an increased interest in investigating the involvement of bone metabolism pathways such as Wnt pathways in aortic valve calcification.^6^ Canonical Wnt signaling has previously been implicated in human valve calcification,^7^ however, mounting evidences demonstrated that the noncanonical Wnt ligands Wnt5a, Wnt5b, and Wnt11 also play essential roles in osteogenesis, making them critical targets in the research of aortic valve calcification.^8^

Enoyl coenzyme A hydratase 1 (ECH1) was shown to be a critical component in mitochondrial fatty acid ‐oxidation.^9^,In recent years, several studies have revealed that ECH1 not only regulates lipid metabolism,^10^ but also participates in the biological behaviors of tumor lymphatic metastasis and the regulation of hepatocarcinoma cells.^11^ Simultaneously, ECH1 was discovered to be a potential biomarker for the early identification of lung cancer^12^ and Alzheimer's Disease.^13^ Nonetheless, the role of ECH1 in CAVD remains unknown.

In this study, we used single‐cell sequencing to discover that ECH1 was primarily expressed in hVICs in human calcific aortic valves (CAVs). To investigate the effects of ECH1 overexpression on aortic valve calcification, ApoE^−/−^ mice fed a normal diet (ND) or a high cholesterol diet (HCD) were infected with adeno‐associated virus serotype 2 (AAV2) containing ECH1 vector. Overexpression of ECH1 reduced aortic valve calcification in ApoE^−/−^ mice treated with HCD, as evidenced by decreased aortic valve leaflet thickness and calcium deposition, improved echocardiographic parameters (decreased peak transvalvular jet velocity and mean transvalvular pressure gradient, as well as increased aortic valve area), and decreased levels of osteogenic markers (Runx2 and osteocalcin) in the aortic valves. During ECH1 expression variations, we also observed modifications in the noncanonical Wnt signaling pathway. Therefore, it is important to clarify the exact function of noncanonical Wnt signaling as well as whether it contributes to ECH1‐mediated osteogenic differentiation. CHIR99021, a Wnt agonist,^14^ can substantially reduce the progression of valve calcification caused by ECH1 knockdown. B, Chromatin immunoprecipitation (ChIP) indicated that ECH1 knockdown enhanced Runx2 transcriptional regulation on downstream target genes, indicating that ECH1 may regulates valve calcification primarily via the noncanonical Wnt signaling pathway.

Our study is the first to reveal the role of Ech1 in valvular calcification. This means that Ech1 may represent a novel therapeutic strategy for CAVD.

## 2 MATERIALS AND METHODS

### 2.1 Ethical statement

Human calcified aortic valve leaflets are obtained from patients with CAVD during aortic valve replacement. Healthy aortic valve tissue specimens were collected from patients who underwent aortic coarctation repair requiring aortic valve replacement. Bicuspid aortic valves, valves with moderate‐to‐severe aortic valve regurgitation, infective endocarditis, congenital valve disease, and rheumatic aortic valvulopathy were all excluded. This study's procedure adhered to the Helsinki Declaration and was approved by the Tongji Medical College Institutional Review Board at Huazhong University of Science and Technology. All patients provided written informed consent.

### 2.2 Single‐cell RNA sequencing

Single‐cell gene expression profiles from 6 aortic valve samples were processed according to the Demonstrated Protocol (Document CG00055, 10X genomics) by Chromium Single Cell 3’ (v2 Chemistry). Approximately 2 × 10^4^ live cells were used to generate single‐cell gel‐beads in emulsion. After reverse transcription, gel‐beads in emulsion were disrupted. Single Cell 3′ v2 libraries were generated following the Single Cell 3′ v2 Reagent Kits User Guide (Document CG00052, 10X genomics). The libraries were sequenced on the Illumina HiSeq Xten platform as previously described.^15^ All data and materials have been made publicly available at the BioProject database of National Center for Biotechnology Information and can be accessed at PRJNA562645 for single‐cell sequencing raw data, at PRJNA552159 for Bulk sequencing raw data.

### 2.3 Animal studies and AAV2 construction

ApoE^−/−^ (C57BL/6 background) mice were housed in a controlled environment (20 ± 2°C, 12‐h/12‐h light/dark cycle) and have free access to water and diet. The animal protocol was reviewed and approved by The Institutional Animal Research Committee of Tongji Medical College.

Adeno associated virus subtype 2 encoding murine ECH1 (AAV2‐ECH1) or short‐hairpin RNA (shRNA) targeting ECH1 (AAV2‐shECH1) delivered into 4‐week‐old ApoE^−/−^ mice through the tail vein 4 weeks prior to diet treatment, AAV2‐Empty and AAV2‐scramble (AAV2‐scr) was performed as negative control respectively. The adeno‐associated viral vectors were designed and provided by OBiO Technology Corp., Ltd.

ApoE^−/−^ mice (8 weeks old) were randomly allocated into the following groups and observed for 24 weeks: mice fed a ND and administered AAV2‐ECH1 or AAV2‐shECH1 (*n* = 10); mice fed an ND and administered AAV2‐Empty or AAV2‐scr (*n* = 10); mice fed a 0.25% high cholesterol diet (HCD; TD 88137, Harlan Teklad, Madison.) and administered AAV2‐ECH1 or AAV2‐shECH1 (*n* = 10); and mice fed an HCD and administered AAV2‐Empty or AAV2‐scr (*n* = 10). HCD‐fed animals were maintained on the diet for 24 weeks to induce aortic valve calcification.

Mice were sacrificed after final transthoracic echocardiography and hemodynamic evaluation, and aortic valves were taken for histological investigation.

### 2.4 Echocardiography

Transthoracic echocardiography was conducted at the end of the experiment under 2.5% isoflurane anesthesia. The images were captured with a Vevo 1100 Imaging system and an 18–38 MHZ phased‐array probe (MS400). Continuous wave Doppler was used to calculate transvalvular velocity. A skilled operator who was not aware of the assignments recorded the echocardiographic data.

### 2.5 Immunobloting

Protease and phosphatase inhibitor combinations were used to homogenize the tissue samples and cells in RIPA Lysis. The protein samples were put onto 4%–20% SDS‐PAGE gels and then, using a wet‐transfer method, transferred onto nitrocellulose membrane. Membranes were treated with the following primary antibodies (Runx2, 12556S, Cell Signaling Technology; Osteocalcin, ab93876, Abcam; Wnt5a, ab220200, Abcam; ECH1, ab153720, Abcam; *β*‐tubulin, ab6046, Abcam) at 4°C overnight, followed by incubation with the horseradish peroxidase (HRP)‐conjugated secondary antibody for 1 h, after blocking with 5% skim milk in TBS‐T (50 mM Tris/HCL, pH 7.6, 150 mM NaCl, and 0.1% Tween‐20) at room temperature for 1 h. The technique for improved chemiluminescence was used to create membranes. Protein loading was normalized using *β*‐actin. The Image J program (Wayne Rasband, National Institutes of Health, Bethesda, MD) from the National Institutes of Health was used to examine band density.

## 3 QUANTITATIVE REAL‐TIME PCR

Total RNA was isolated from aortic valve tissues of mice and human VICs by using RNAiso Plus (Takara) according to the manufacturer's instructions. Reverse transcription of RNA was performed using PrimeScript™ real time Master Mix (Takara). Quantitative real‐time PCR was performed using SYBR_Premix Ex Taq™ (Takara) on a StepOnePlus™ Real‐time PCR System (Applied Biosystems, Foster City, CA). Relative RNA levels were calculated using the comparative threshold cycle (2‐ΔΔCt) method. The sequences of primers used in the present study are as follows: Wnt5a‐Forward: 5ʹ‐CAACTGGCAGGACTTTCTCAA‐3ʹ, Wnt5a ‐Reverse: 5ʹ‐CATCTCCGATGCCGGAACT‐3ʹ; Wnt5b‐Forward: 5ʹ‐CTGCTGACTGACGCCAACT‐3ʹ, Wnt5b‐Reverse: 5ʹ‐CCTGATACAACTGACACAGCTTT‐3ʹ; Wnt11‐Forward: 5ʹ‐GCTGGCACTGTCCAAGACTC‐3ʹ, Wnt11‐Reverse: 5ʹ‐CTCCCGTGTACCTCTCTCCA‐3ʹ; osteopontin (OPN)‐Forward: 5ʹ‐AGCAAGAAACTCTTCCAAGCAA‐3ʹ, OPN‐Reverse: 5ʹ‐GTGAGATTCGTCAGATTCATCCG‐3ʹ; BMP2‐Forward: 5ʹ‐GGGACCCGCTGTCTTCTAGT‐3ʹ, BMP2‐Reverse: 5ʹ‐TCAACTCAAATTCGCTGAGGAC‐3ʹ; GAPDH‐Forward: 5ʹ‐AGGTCGGTGTGAACGGATTTG‐3ʹ, GAPDH‐Reverse: 5ʹ‐TGTAGACCATGTAGTTGAGGTCA‐3ʹ; ECH1‐Forward: 5ʹ‐GCTACCGCGATGACAGTTTC‐3ʹ, ECH1‐Reverse: 5ʹ‐TCAGAGATCGAAGGCTGATGTT‐3ʹ.

### 3.1 Immunofluorescence staining

Immunofluorescence staining was used to identify Runx2 in the mouse aortic valve leaflets. Frozen portions of aortic valves were dried at room temperature for 15 min, then fixed in 4% paraformaldehyde (PFA) for 20 min, followed by permeabilization with 0.1% Triton X‐100 in phosphate‐buffered saline (PBS) for an additional 20 min. After that, the tissues were incubated with the primary antibody against ECH1 (Abcam, Cambridge), followed by incubation with fluorescently conjugated secondary antibody (Abcam) and counterstaining with 4′,6‐diamidino‐2‐phenylindole (DAPI) (Sigma‐Aldrich).

### 3.2 Calcium assay

The calcium assay was performed as previously described.^16^ The calcium concentration in aortic valve cusp extracts prepared from 0.1 mol/L hydrochloric acid was calculated colorimetrically using the o‐cresolphthalein technique. The calcium concentration was represented as calcium per milligram of weight and normalized to either the weight of the cusps on the aortic valve or the total protein of the cell.

### 3.3 Alizarin red staining

The rehydrated paraffin slices were rinsed with PBS and stained with the Alizarin Red staining for 5 min at room temperature. The dark red nodules were recognized as positive staining after three washes with 95% ethanol to eliminate non‐specific staining. Images were captured with a Leica digital microscope. Quantification was performed by using Image‐Pro Plus software.

Cells were cultured with appropriate interventions in a conditioning medium (growth medium supplemented with 10 mmol/L *β*‐glycerophosphate, 10 nmol/L dexamethasone, 4 μg/mL cholecalciferol, and 8 mmol/L CaCl2) for 21 days in order to analyze calcium deposition in AVICs. Three days a week, the media was switched. Calcium deposit detection was accomplished using Alizarin Red S staining. In brief, cells were fixed in 4% paraformaldehyde for 15 min after being cleaned with PBS. Following a half‐hour incubation period with a 0.2% alizarin red solution (pH 4.0–4.2), surplus dye was eliminated using a distilled water wash. Alizarin red stains were bleached with 10% acetic acid at 75°C in order to measure the calcium deposition.

### 3.4 Luciferase reporter assays

Using the One Step Cloning Kit (C112‐02; Vazyme Biotech Ltd.), Promoter of Runx2 was subcloned into the pGL3‐ Basic vector (Promega.). In MC3T3‐E1 cells, Renilla luciferase reporter plasmid (pRL‐TK; Promega) was co‐transfected with luciferase reporter constructs before different treatments. The Dual Luciferase Reporter Assay Kit (Promega) was then used to measure the luciferase activity after the cells had been collected and lysed, in accordance with the manufacturer's instructions.

### 3.5 Chromatin immunoprecipitation (ChIP) assay

A Magna ChIP One‐Day Chromatin Immunoprecipitation Kit (#17‐100859; Millipore/Upstate.) was used for ChIP assays, following the manufacturer's instructions. A total of 1 × 10^6^ MC3T3‐E1 cells were prepared for chromatin immunoprecipitation using anti‐Runx2 (12556S; Cell Signaling Technology) or anti‐rabbit IgG (ABclonal Biotechnology.) antibodies. Quantitative polymerase chain reaction was performed to analyze the precipitated genomic DNA using primer sets targeting OPN and Osteocalcin enhancers. The sequences of the primers used for ChIP are as follows: OPN promoter, forward primer sequence 5′‐ACTGTAGATTGTGTGTGTGC‐3′; reverse primer sequence: 5′‐ CTGCCTCCTCCTGCTGCTGC‐3′; Osteocalcin promoter, forward primer sequence 5′‐TCGTCCACTCCCAGAGCCTTGC‐3′; reverse primer sequence: 5′‐CTGCACCCTCCAGCATCCAG‐3′.

### 3.6 Determination of total cholesterol and triglyceride levels

Animals underwent a 14–15 h fast before blood samples were collected. According to the manufacturer's instructions, the manufacturer's specified kits from Biosino, China, were used to measure the levels of total cholesterol (TC) and triglycerides (TG).

### 3.7 Statistical analyses

All statistical analyses were performed with GraphPad Prism 9.0 with data points denoting mean ± SD. Each series of experiments was repeated at least three times. When comparing two groups, a student's *t*‐test was used, and if comparing multiple groups, a one‐way analysis of variance was used, followed by Bonferroni post‐hoc testing. The specific statistical methods are listed in detail in figure legends. The corresponding *p*‐value is shown in the figures.

## 4 RESULTS

### 4.1 ECH1 is down‐regulated in human calcific aortic valves

**FIGURE 1:**
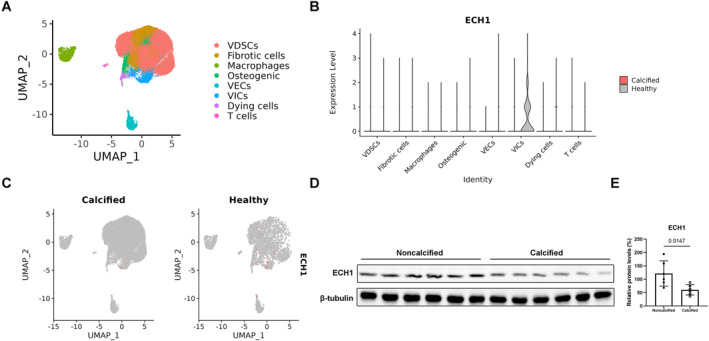
Enoyl coenzyme A hydratase 1 (ECH1) is down‐regulated in human calcified aortic valves|calcific aortic valves. Single‐cell sequencing analysis of 6 aortic valve specimens (2 healthy and 4 calcified aortic valve lesions [calcific aortic valve disease] aortic valves), with a total of approximately 35,000 cells detected. (A) Cell subpopulation analysis. (B) Distribution of Ech1 in different cell subpopulations. (C) Expression and distribution of Ech1 (marked red) in normal and calcified valve cells. (D–E) The protein levels of ECH1 in aortic valve leaflets, unpaired two‐tailed Student's *t*‐test, *n* = 6 per group.

Six patients were selected for aortic valve leaflet collection based on ultrasonography as previously described.^15^ After surgery, each of the 6 aortic valve specimens (2 healthy, 4 CAVD) was digested to obtain single cell suspensions which were analyzed by scRNA‐seq (Figure 1A). Analysis of different cell subpopulations in the valve revealed that Ech1 was predominantly expressed in interstitial valve cells and was significantly reduced in CAVs (Figure 1B–C, marked red). Immunoblots also indicated that the protein level of ECH1 was decreased in CAVs (Figure 1D–E). These data suggested that ECH1 is down‐regulated in human CAVs.

### 4.2 Ech1 overexpression alleviates HCD induced aortic valve calcification

**FIGURE 2:**
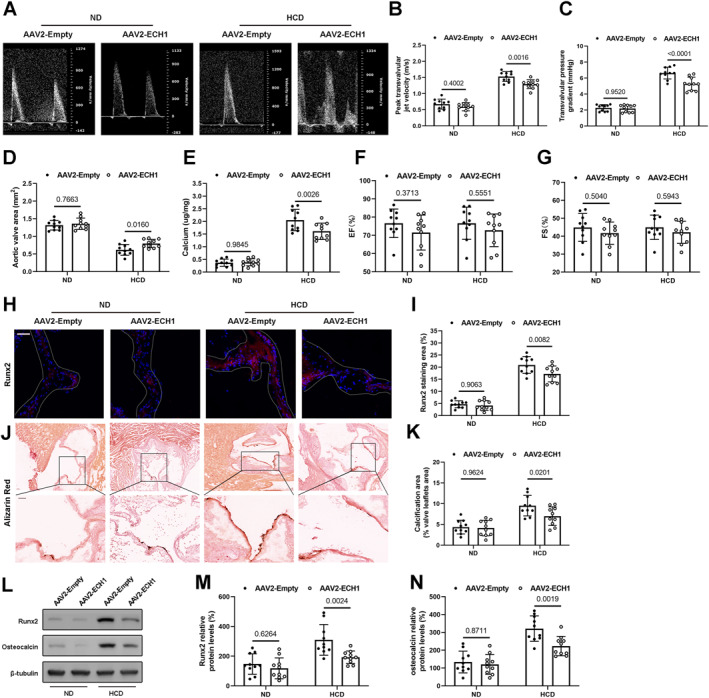
Enoyl coenzyme A hydratase 1 (ECH1)overexpression reduces aortic valve calcification in vivo. (A) Echocardiographic data of experimental mice. (B) Peak transvalvular jet velocity, (C) mean transvalvular pressure gradient, (D) Aortic valve area, (F) Ejection Fractions (EF%) and (G) Fraction Shorting (FS%). (E) The calcium content of aortic valve leaflets. (H) Immunofluorescence assay of Runx2 (Red) in aortic valve leaflets and its statistical results (I). J, Representative images calcium deposits (alizarin red) in mice of different groups and its statistical results (K). (L–M) The protein levels of osteogenic markers (Runx2 and osteocalcin) in aortic valve leaflets. Two‐way ANOVA followed by Bonferroni post hoc test. *n* = 10 per group. Scale bar: 50 um. Values are the mean ± SD. ANOVA, analysis of variance.

To investigate the role of ECH1 in aortic valve calcification in vivo, we used AAV2‐ECH1 (or AAV2‐empty control) to overexpress ECH1 in ApoE^−/−^ mice by tail vein injection.^17^ ECH1 overexpression in the aortic valves was found to be effective (Figure S1A–C). After 24 weeks, mice in the HCD AAV2‐scr group displayed significantly higher mean transvalvular pressure gradient and peak transvalvular jet velocity, as well as significantly lower aortic valve area (Figure 2A–D). These anomalies were somewhat corrected by overexpressing ECH1, although mouse heart function was unaffected(Figure 2F–G). The morphology and calcification of valve leaflets in ApoE−/− mice were next analyzed. In ApoE−/− mice, HCD induced aortic valve calcification as shown by elevated aortic valve Runx2 expression (Figure 2H–I). By using alizarin red staining and detecting calcium deposition, we discovered sizable calcium deposits in the AoV leaflets of ApoE−/− animals, which were clearly decreased by ECH1 overexpression (Figure 2E,J–K). Similarly, ECH1 overexpression lowered the levels of osteogenic markers and largely reversed these alterations (Figure 2L–N). However, TC and triglyceride levels were not significantly altered by ECH1 overexpression (Figure S2A–B). These findings imply that overexpression of ECH1 reduces aortic valve calcification in ApoE−/− mice rather than by affecting blood lipid levels in mice.

### 4.3 ECH1 silencing accelerates aortic valve calcification in vivo

**FIGURE 3:**
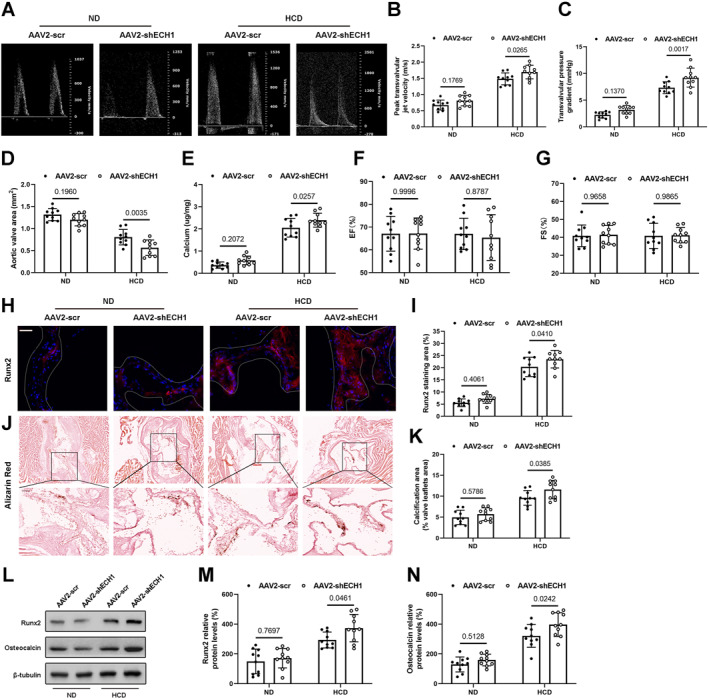
Enoyl coenzyme A hydratase 1(ECH1) deletion aggravates aortic valve calcification in vivo. (A) Echocardiographic data of experimental mice. (B) Peak transvalvular jet velocity, (C) mean transvalvular pressure gradient, and (D) Aortic valve area, (F) Ejection Fractions (EF%) and (G) Fraction Shorting (FS%). (E) The calcium content of aortic valve leaflets. H, Immunofluorescence assay of Runx2 (Red) in aortic valve leaflets and its statistical results (I). J, Representative images calcium deposits (alizarin red) in mice of different groups and its statistical results (K). (L–M) The protein levels of osteogenic markers (Runx2 and osteocalcin) in aortic valve leaflets. Two‐way ANOVA followed by Bonferroni post hoc test. *n* = 10 per group. Scale bar: 50 um. Values are the mean ± SD. ANOVA, analysis of variance.

We also used rat tail injection of AAV2‐shECH1 to diminish ECH1 expression and further clarify the function of ECH1 in aortic valve calcification. The effectiveness of ECH1 silencing in aortic valves was demonstrated (Figure S1D‐F). In contrast to ECH1 overexpression, ECH1 knockdown worsened aortic valve calcification associated with the 24‐week HCD diet, and we found an increase in transvalvular pressure gradients (Figure 3A–C), a reduction in aortic valve area (Figure 3D), an increase in calcium deposits (Figure 3J–K) and Runx2 (Figure 3H–I) and its downstream target gene expression in aortic valve leaflets (Figure 3L–N), and increased calcium deposition (Figure 3E). Nevertheless, we also found no discernible alterations in the heart function of the mice (Figure 3F–G). These data imply that knockdown of ECH1 worsens aortic valve calcification in vivo but does not impact cardiac function.

### 4.4 Evidence of Wnt5a involvement in the regulation of aortic valve calcification by ECH1

**FIGURE 4:**
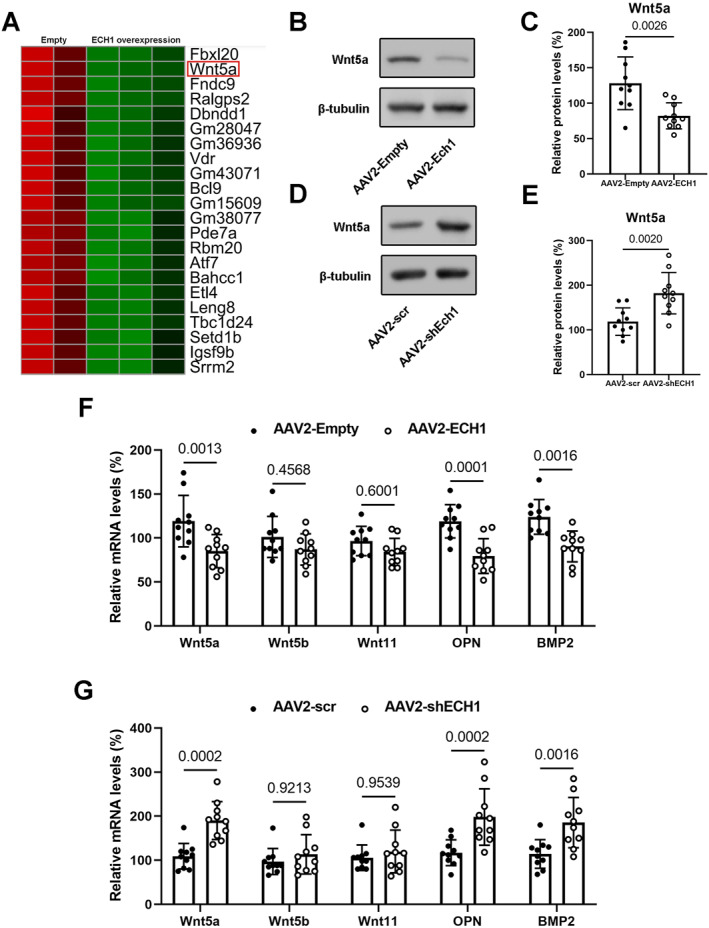
Changes in the non‐classical Wnt pathway during the regulation of Enoyl coenzyme A hydratase 1 (ECH1) expression. (A) Heatmap of differentially expressed genes when human aortic valve interstitial cells transfected with ECH1 plasmid or empty vector. (B–E) Western blot analysis for Wnt5a in the aortic valve leaflets of experimental mice. (F–G) Relative mRNA levels of non‐classical Wnt ligands (Wnt5a, Wnt5b and Wnt11) and osteogenic markers (OPN and BMP2) in mice aortic valve leaflets. Unpaired two‐tailed Student's *t*‐test. *n* = 10 per group. Values are the mean ± SD. OPN, osteopontin.

To elucidate the underlying mechanism, we carried out transcriptome analysis of hVICs transfected with ECH1 plasmid or empty vector. The differentially expressed genes were depicted in a heatmap, which indicated that ECH1 overexpression significantly reduced Wnt5a mRNA levels (Figure 4A). As previously reported, the noncanonical Wnt ligands Wnt5a, Wnt5b and Wnt11 exerted critical roles in osteogenesis, which made them key targets for the study of aortic valve calcification.^8^ Therefore, we examined the relative protein expression of Wnt5a in CAVs in the presence of ECH1 overexpression and silencing, protein levels of Wnt5a are reduced in the presence of ECH1 overexpression but increased in the presence of ECH1 knockdown (Figure 4B–E). We also examined the mRNA expression of Wnt5a, Wnt5b and Wnt11 in the aortic valve by RT‐PCR and only found significant alteration of Wnt5a mRNA levels. Meanwhile, the changes in calcification‐related genes OPN, BMP2 were consistent with Wnt5a (Figure 4F–G). Therefore, ECH1 inhibits Wnt5a and attenuates aortic valve calcification.

### 4.5 Ech1 regulates transcriptional activity of runx2 via altered WNT5a/Ca^2+^ signaling

**FIGURE 5:**
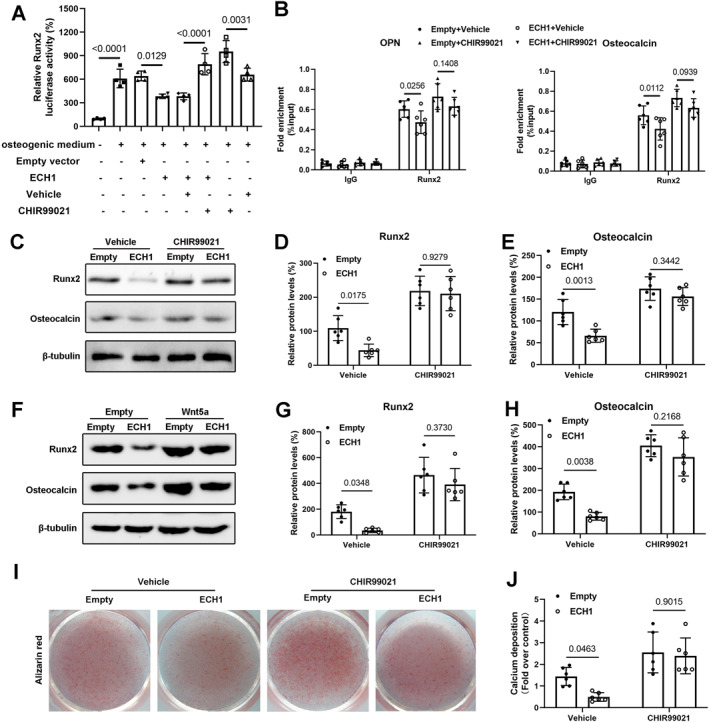
Enoyl coenzyme A hydratase 1 (ECH1) regulates transcriptional activity of RUNX2 via altered Wnt5a/Ca^2+^ Signaling. (A) Luciferase (Luc) reporter constructs containing promoters of Runx2 were co‐transfected with an internal control plasmid pRL‐TK into MC3T3‐E1 cells, with or without treatment of osteogenic medium and CHIR99021 (Wnt agonist). One‐way ANOVA followed by Bonferroni post hoc test. *n* = 4 per group. (B) Chromatin immunoprecipitation assay was performed in MC3T3‐E1 cell line. IgG was used as a negative control. (C–H) Immunoblots for Runx2 and Osteocalcin in osteogenic medium treated human aortic valve interstitial cells (hVICs) overexpressing ECH1, followed by different treatments and statistical analysis. I–J, Alizarin red staining of mineralization nodules in hVICs with different treatment. Two‐way ANOVA followed by Bonferroni post hoc test. *n* = 6 per group. Two‐way ANOVA followed by Bonferroni post hoc test. *n* = 6 per group. Values are the mean ± SD. ANOVA, analysis of variance.

Runx2 is an important transcription factor in hVICs osteogenic differentiation, and it can directly stimulate transcription of osteogenic‐related genes like OPN and Osteocalcin by binding to specific enhancer regions.^18^, ^19^, ^20^, ^21^ By luciferase reporter assay, we found osteogenic medium treatment significantly enhanced the promoter activity of Runx2, while this effect was blunted by ECH1 overexpression, suggesting that ECH1 inhibited Runx2 transcription. However, upon CHIR99021 (Wnt signaling agonist) treatment, the promoter activity of Runx2 inhibited by ECH1 was re‐activated, indicating that ECH1 inhibited Runx2 transcription via reducing Wnt5a signaling (Figure 5A). Subsequently, ChIP experiments were performed to further determine the effect of ECH1 on the DNA binding activity of Runx2. Our results showed that ECH1 overexpression inhibited the binding of Runx2 to its downstream gene promoters, including OPN and Osteocalcin, however, CHIR99021 administration eliminated the inhibitory role of ECH1 on Runx2 binding activity to OPN and Osteocalcin (Figure 5B). Western blot also revealed that ECH1 attenuated the expression of RUNX2 and Osteocalcin proteins in hVICs in the presence of calcification medium treatment, but the protective effect of ECH1 overexpression in attenuating calcification‐related gene expression was was lessened when CHIR99021 treatment (Figure 5C–E) or wnt5a overexpression (Figure 5F–H) was present. Furthermore we observed that ECH1 reduced calcium deposition in hVICs stimulated by calcification medium, but this protective effect was significantly attenuated in the presence of CHIR99021 (Figure 5I–J). Collectively, ECH1 regulates transcriptional activity of Runx2 via altered Wnt5a/Ca^2+^ signaling, which in turn regulates valvular calcification.

## 5 DISCUSSION

CAVD is a complex disease with significant spatiotemporal heterogeneity,^22^ with inherited variables, lipid deposition, inflammation, and osteogenic transformation of heart VICs all playing roles in its development.^23^ Despite the identification of a few risk factors, the molecular processes underlying aortic valve calcification remain unknown. There are currently no effective therapies to prevent CAVD development.

This is the first study to describe the involvement of ECH1 in valve calcification. We discovered that decreased ECH1 levels in human aortic valves were relevant to valve calcification. ECH1 overexpression mitigated aortic valve calcification, but ECH1 silencing had the reverse effect. Notably, we also identified that Wnt5a, a noncanonical Wnt ligand, was also altered when ECH1 expression was modulated. Furthermore, we discovered that ECH1 exerted anti‐calcific actions through reducing Wnt signaling, since CHIR99021, a Wnt agonist, may significantly lessen the protective impact of ECH1 overexpression on the development of valve calcification. The ChIP and Luciferase assays all showed that ECH1 overexpression prevented Runx2 binding to its downstream gene promoters, including OPN and osteocalcin, due to calcified medium treatment, while CHIR99021 neutralized this protective effect. These results point to a heretofore unknown regulatory ECH1‐Wnt5a axis that contributes to CAVD.

Wnt signaling pathways include classical or canical *β*‐catenin dependent pathways and *β*‐catenin independent pathways, which are collectively referred to as noncanical pathways.^24^ Wnt signaling pathways regulate endothelial‐to‐mesenchymal transition and cell proliferation during the early phases of endocardial cushion and heart valve development. It is widely established that canonical Wnt signaling is linked to valve calcification. The importance of the noncanonical Wnt signaling pathway in valve calcification has steadily been recognized in recent years.^25^ The mRNA level of Wnt5a was dramatically regulated when ECH1 expression was varied in the current study. However, no substantial difference in Wnt5b and Wnt11 alterations was identified.

Much evidence suggests that ECH1 regulates nonalcoholic fatty liver disease, type 2 diabetes,^26^ and obesity.^27^ However, its involvement in CAVD remained unknown. Here, we demonstrated for the first time that ECH1, which is mostly expressed in interstitial valve cells, was decreased in human CAVs and tend to slow aortic valve calcification development in vivo. We examined liver lipid levels in mice since ECH1 influences lipid metabolism but found no statistically significant alterations, it seems that overexpression of ECH1 did not significantly improve the lipid profile of mice on a HCD, which can be further investigated later. Thus, in the present investigation, the protective effect of ECH1 overexpression was not dependent on the regulation of blood lipid levels in mice.

There are several limitations to this study. First, the sample size of our study is limited due to the value of clinical samples and the difficulty in collecting them. Second, we investigated the role of ECH1 in CAVD, but why it is decreased in calcified valves and what its upstream regulatory mechanisms are still unknown. Third, we did not adjust for gender differences.^28^, ^29^, ^30^ To effectively address this issue, both males and females should be included, and data should be analyzed independently by sex. We can do further studies to explore the aforementioned questions in the follow‐up study.

In conclusion, this study demonstrated for the first time that ECH1 overexpression attenuates aortic valve calcification in ApoE^−/−^ mice. ECH1 overexpression reduced aortic valve calcification both in vitro and in vivo. Mechanistically, we have also established a link between ECH1 and nonconical Wnt signaling in hVICs, ECH1 inhibited the osteogenic differentiation of hVICs in aortic valve calcification by suppressing Wnt5a (Graphical abstract). Therefore, our study provides novel insights into the molecular mechanism regulating Wnt5a/Ca^2+^ in the pathogenesis of CAVD, targeting ECH1 may prove of therapeutic potential to aortic valve calcification.

## AUTHOR CONTRIBUTIONS

Caijun Rao and Baoqing Liu conceived and designed the experiments. Caijun Rao and Baoqing Liu performed the experiments. Haojie Qin analyzed the data and contributed reagents/materials/analysis tools. Zhipeng Du and Caijun Rao drafted the manuscript. All authors read and approved the final manuscript.

## CONFLICT OF INTEREST STATEMENT

None.

## ETHICS STATEMENT

The Institutional Review Board of Huazhong University of Science and Technology has approved our research procedures.

## CONSENT FOR PUBLICATION

Yes.

## Supporting information

Supporting Information S1

## Data Availability

Data are available from the corresponding author upon reasonable request.
