# Supplementary material for: Enoyl coenzyme a hydratase 1 attenuates aortic valve calcification by suppressing Runx2 via Wnt5a/Ca2+ pathway
Source: J Cell Commun Signal. 2024 May 31;18(2):e12038. doi: 10.1002/ccs3.12038 (PMC11208118; doi:10.1002/ccs3.12038)
Supplement: Supplementary file 1 — Supporting Information S1 [file CCS3-18-e12038-s001.docx]

**Supporting information**

**Supplemental figure**

**
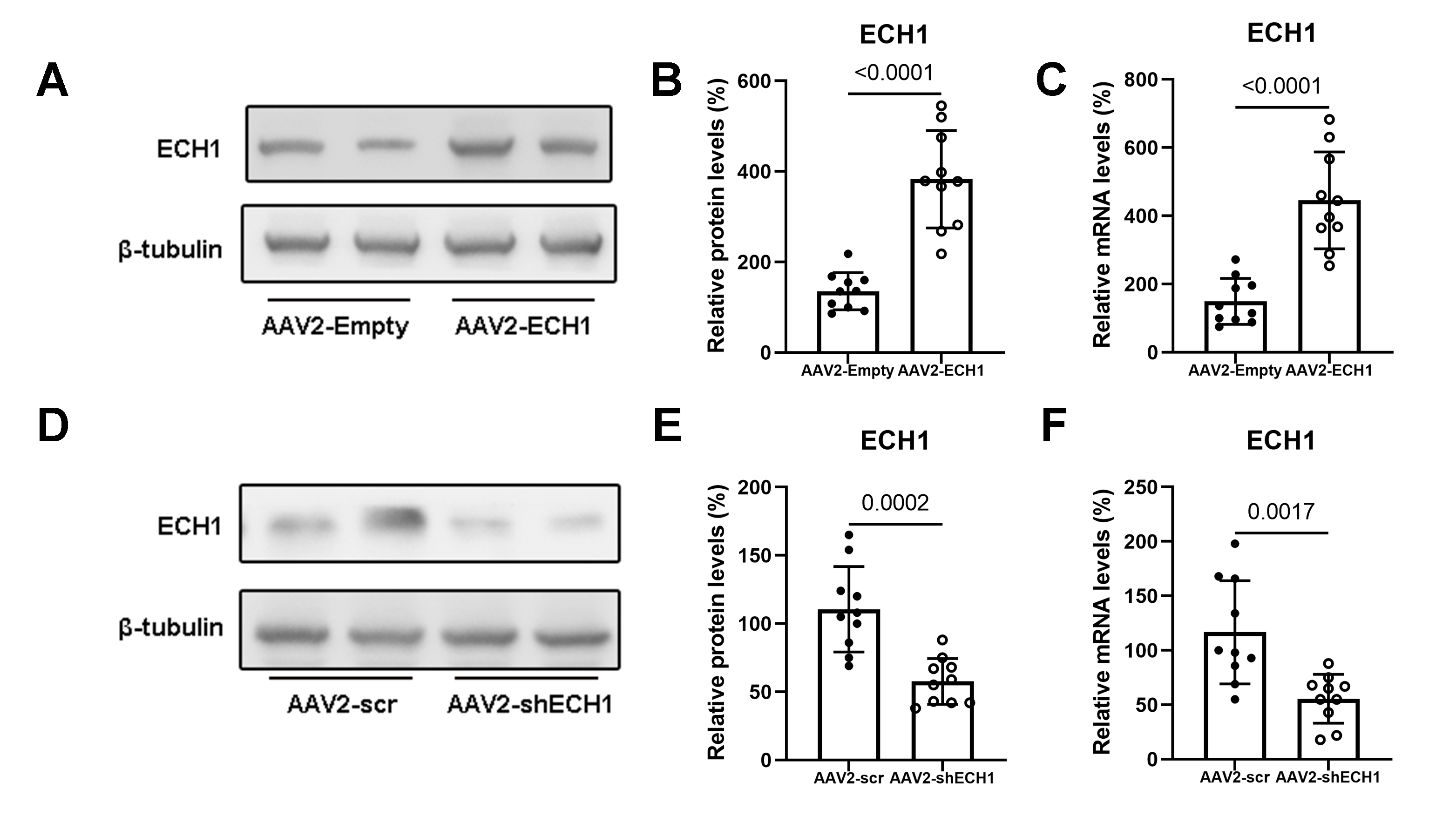
**

**Figure S1 Relative expression level of ECH1 protein and mRNA in mice aortic valve leaflets.** Unpaired two-tailed Student’s t-test. n= 10 per group. Values are the mean ± SD.


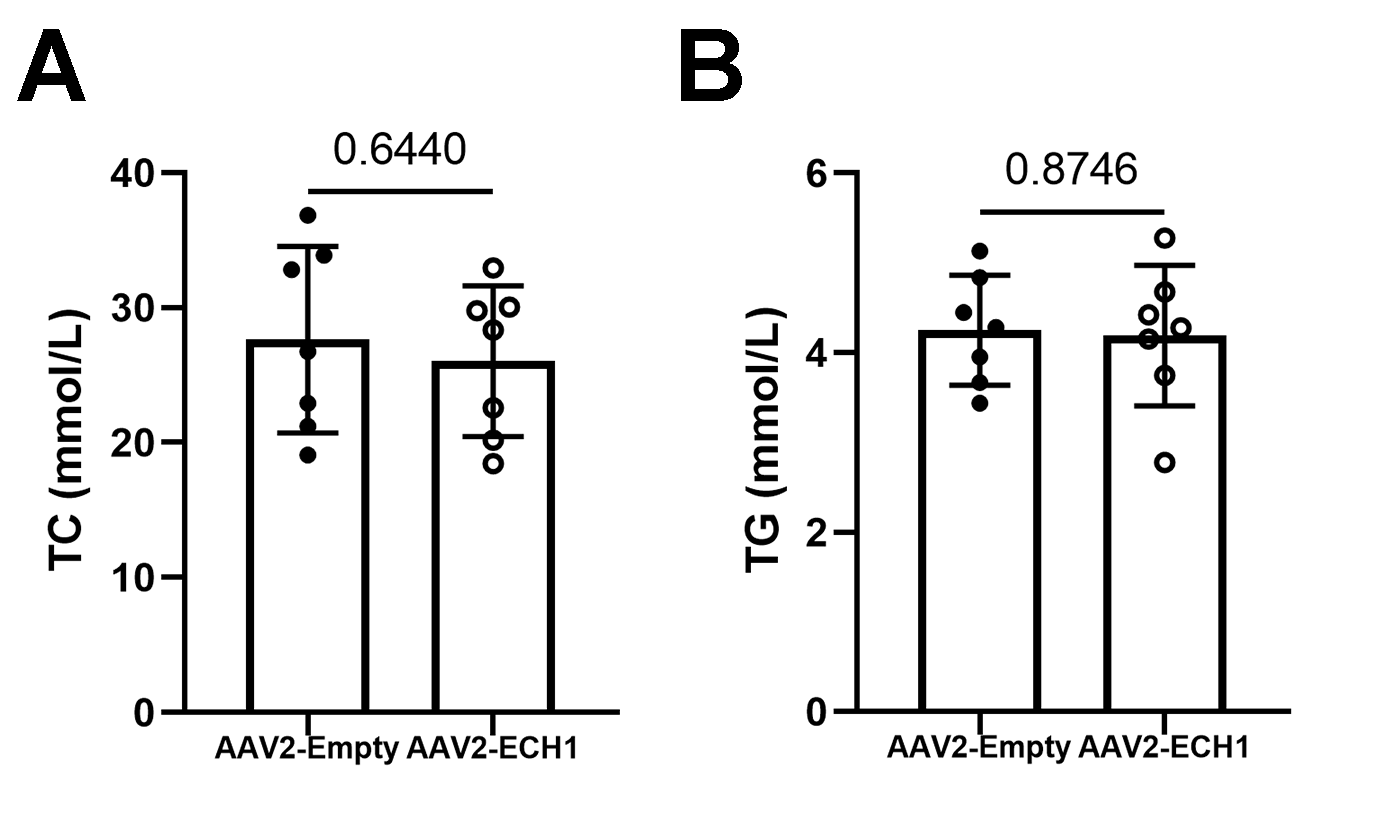


**Figure S2 Metabolic parameters in different group of mice fed with high cholesterol diet for 24 weeks.** Unpaired two-tailed Student’s t-test. n= 7 per group. Values are the mean ± SD.
